# The role of lactate-induced protein lactylation in gliomas: implications for preclinical research and the development of new treatments

**DOI:** 10.3389/fphar.2024.1383274

**Published:** 2024-06-25

**Authors:** Xiaoying Liu, Yue Zhou, Haichuan Wang

**Affiliations:** ^1^ Department of Pharmacy, Xindu District People’s Hospital of Chengdu, Chengdu, China; ^2^ Department of Paediatrics, Sichuan Academy of Medical Science and Sichuan Provincial People’s Hospital, School of Medicine, University of Electronic Science and Technology of China, Chengdu, China

**Keywords:** lactylation, gliomas, lactate, epigenetic regulation, cancer therapy

## Abstract

The most prevalent primary brain tumors in adults are gliomas. In addition to insufficient therapeutic alternatives, gliomas are fatal mostly due to the rapid proliferation and continuous infiltration of tumor cells into the surrounding healthy brain tissue. According to a growing body of research, aerobic glycolysis, or the Warburg effect, promotes glioma development because gliomas are heterogeneous cancers that undergo metabolic reprogramming. Therefore, addressing the Warburg effect might be a useful therapeutic strategy for treating cancer. Lactate plays a critical role in reprogramming energy metabolism, allowing cells to rapidly access large amounts of energy. Lactate, a byproduct of glycolysis, is therefore present in rapidly proliferating cells and tumors. In addition to the protumorigenesis pathways of lactate synthesis, circulation, and consumption, lactate-induced lactylation has been identified in recent investigations. Lactate plays crucial roles in modulating immune processes, maintaining homeostasis, and promoting metabolic reprogramming in tumors, which are processes regulated by the lactate-induced lactylation of the lysine residues of histones. In this paper, we discuss the discovery and effects of lactylation, review the published studies on how protein lactylation influences cancer growth and further explore novel treatment approaches to achieve improved antitumor effects by targeting lactylation. These findings could lead to a new approach and guidance for improving the prognosis of patients with gliomas.

## Introduction

The most common primary brain tumors are gliomas. Gliomas account for approximately 30% of all cancers of the central nervous system and 80% of all malignant brain tumors. The ability to infiltrate the surrounding normal brain tissue is a distinguishing characteristic of these tumors (Goodenberger and Jenkins, 2012). Gliomas are now categorized according to the cell type with which they share histological traits. Because of their similarity to astrocytes, oligodendrocytes, ependymal cells, and mixed glial cells, gliomas are designated astrocytomas (multiform glioblastoma), oligodendrogliomas, ependymomas, and mixed gliomas (oligoastrocytomas), respectively. The World Health Organization categorizes gliomas into grades I through IV, with a higher grade denoting a more malignant tumor. Grade III and Grade IV gliomas are malignant tumors with a high rate of proliferation (Grade III) and angiogenic activity (Grade IV, glioblastoma), and Grade I and Grade II gliomas are slower-growing, less aggressive tumors. The most common type of malignant primary brain tumor is malignant glioma (Louis, 2006; Ricard et al., 2012), the prevalence of which has increased during the past few years to nine per 100,000 people (Rasmussen et al., 2017; Davis et al., 2020). Malignant gliomas occur at the highest frequencies among the adult population older than 45 years; however, younger people may also be impacted by this incredibly aggressive tumor (Porter et al., 2010). Because malignant gliomas proliferate rapidly and permeate the surrounding brain tissue (Cheng et al., 2011; Agosti et al., 2024), the treatment and prognosis of gliomas remain dismal (Table 1). Consequently, it is critical to investigate the mechanisms underlying the emergence and spread of gliomas and to consider more potent treatment options.

**TABLE 1:** Ongoing clinical trials for gliomas.

Therapy	Phase	Target	Condition	NCT identiers
HMPL-813 (epitinib)	I	growth factor receptors	Not available	NCT03231501 https://clinicaltrials.gov/study/NCT03231501
ERC1671/GM-CSF/Cyclophosphamide + bevacizumab	II	growth factor receptors	Recurrent	NCT01492673 https://clinicaltrials.gov/study/NCT01492673
Pembrolizumab	I	growth factor receptors	Recurrent	NCT02852655 https://clinicaltrials.gov/study/NCT02852655
Veliparib	I	DNA repair and cell cycle control pathways	Newly diagnosed	NCT01026493 https://clinicaltrials.gov/study/NCT01026493
Olaparib	I	DNA repair and cell cycle control pathways	Not available	NCT05252390 https://clinicaltrials.gov/study/NCT05252390
LEE011 (ribociclib)	II	DNA repair and cell cycle control pathways	Recurrent	NCT05429502 https://clinicaltrials.gov/study/NCT05429502
AG-221 (enasidenib)	II	epigenetics and tumor metabolism	Recurrent	NCT02273739 https://clinicaltrials.gov/study/NCT02273739
AG-881 (vorasidenib)	I	epigenetics and tumor metabolism	Not available	NCT05484622 https://clinicaltrials.gov/study/NCT05484622
IDH peptide vaccine	I	epigenetics and tumor metabolism	Not available	NCT05609994 https://clinicaltrials.gov/study/NCT05609994
Lomustine + bevacizumab	II	angiogenesis	Recurrent	NCT01067469 https://clinicaltrials.gov/study/NCT01067469
Topotecan + pazopanib	II	angiogenesis	Recurrent	NCT01931098 https://clinicaltrials.gov/study/NCT01931098
Nivolumab	II	Immunotherapies	Newly diagnosed	NCT03925246 https://clinicaltrials.gov/study/NCT03925246
Dendritic cell vaccine + Temozolomide	I	Immunotherapies	Not available	NCT02649582 https://clinicaltrials.gov/study/NCT02649582
Atezolizumab + Temozolomide	II	Immunotherapies	Newly diagnosed	NCT03174197 https://clinicaltrials.gov/study/NCT03174197

NCT, National Clinical Trail.

## Lactate production and lactylation

**FIGURE 1:**
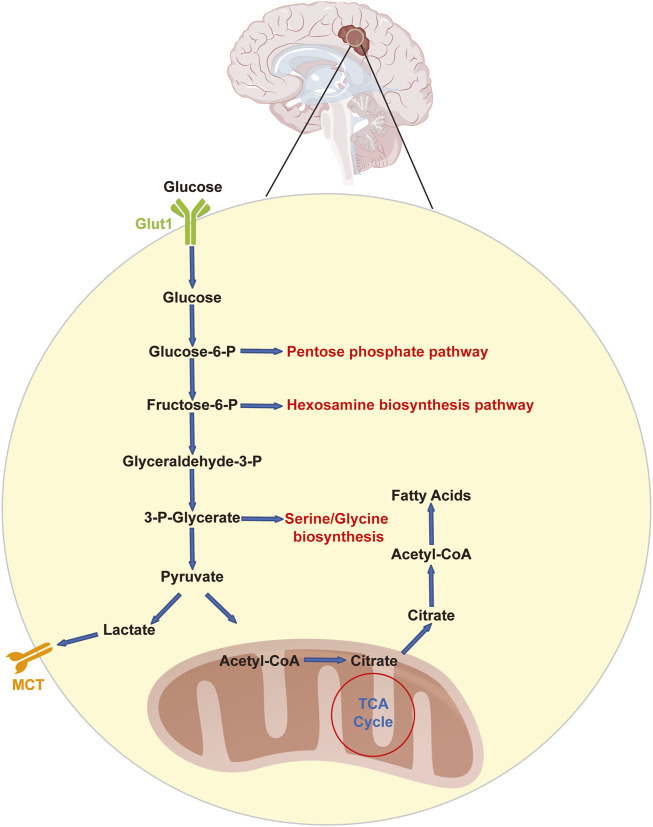
Glycolysis and related pathways in glioblastoma cells. Glucose enters glioblastoma cells via GLUT1 in an insulin-independent manner. Upon entry, glucose is phosphorylated to glucose-6-phosphate and may be processed further in the pentose phosphate pathway or in glycolysis. Glycolysis feeds and communicates with the hexosamine biosynthesis pathway (at the level of fructose-6-phosphate) as well as the serine/glycine pathway (originating from 3-phospho-glycerate). The final product of glycolysis is pyruvate, which can subsequently be converted to lactate, which is removed from the cell through either MCT1 or MCT4. Alternatively, pyruvate can be converted to acetyl-CoA, which in turn reacts with citrate. Citrate can be processed in the TCA cycle or used for the production of cytosolic acetyl-CoA, which is used for fatty acid synthesis. Glucose-6-P: glucose-6-phosphate; fructose-6-P: fructose-6-phosphate; glyceraldehyde-3-P: glyceraldehyde-3-phosphate; LDHA: lactic dehydrogenase; MCT: monocarboxylate transporter; Glut1: glucose transporter 1.

Astrocytes in the normal brain mostly use glycolysis, whereas neurons depend on oxidative phosphorylation (OXPHOS) (Bélanger et al., 2011; Duan et al., 2018). Even in the presence of oxygen, tumor cells exhibit significantly higher glycolytic rates than normal cells. Otto Warburg discovered as early as 1926 that even under aerobic conditions, the uptake of glucose by tumor cells increases rapidly, and tumor cells produce excessive levels of lactate (Warburg et al., 1927). The Warburg effect is a process that has been the subject of much research. Because aerobic glycolysis is less effective than OXPHOS at generating adenosine triphosphate (ATP), scientists have questioned why cancer cells have acquired this energy-producing mechanism (Vander Heiden et al., 2009). Glioblastoma cells metabolize ATP by aerobic glycolysis at an unusually high rate, as proven by lactate generation, which is 20 times greater than the lactate level found in normal tissue, even though ATP is produced less effectively in this mode of metabolism (Duan et al., 2018) (Figure 1). Most notably, the glioblastoma microenvironment is more acidic than that of normal brain tissue because lactate metabolism is closely correlated with extracellular pH (Honasoge and Sontheimer, 2013). In addition, glioblastoma cells may obtain fuel from nearby astrocytes to sustain the high energy levels necessary for rapid multiplication (Bélanger et al., 2011; Brooks, 2018; Jia et al., 2018). Glioblastoma cells absorb metabolites produced by astrocytes, such as lactate, and oxidize these metabolites to provide additional fuel. Thus, lactate buildup is common in solid tumors. Proteome analysis has shown a metabolic transition in glioma cells in response to hypoxia characterized by the activation of all glycolytic pathway enzymes involved in lactate production (Zhang et al., 2017). Similarly, the serum lactate concentration has been proposed as a biomarker for the malignancy grade of gliomas, with high-grade gliomas exhibiting markedly greater serum lactate levels than low-grade gliomas (Wang et al., 2020). Although lactate was long thought to be a “metabolic waste product” of aerobic glycolysis, increasing research has shown that lactate may actually be used as a source of energy by joining the tricarboxylic acid cycle (TCA cycle) and even serving as a multifunctional signaling molecule (De La Cruz-López et al., 2019).

Numerous prevalent posttranslational modifications (PTMs), including phosphorylation, acetylation, methylation, and ubiquitination, have drawn much interest and have been well studied (Zafar et al., 2024). Interestingly, in 2019, a lactate-induced lactylation alteration of histone lysine residues were discovered, and evidence of the function of histone lactylation in carcinogenesis has progressively accumulated (Zhang et al., 2019). From this novel perspective, the high-lactate state of tumor metabolism is linked to carcinogenesis through lactate-induced lactylation, an epigenetic change connected to metabolic stress (Liu et al., 2022; Xin et al., 2022). In this review, we explore the close relationship between recently discovered lactylation modifications and glioblastoma cells in an effort to expand our understanding of tumor epigenetics and metabolism, lay the groundwork for future research into the role this modification plays in the development of gliomas, and provide insight into potential new treatment approaches.

### Mechanisms of lactylation and its writers and erasers

Lactate is a significant byproduct of the Warburg effect and a signaling molecule with nonmetabolic uses, as has been reported in previous research (Chen et al., 2022). Acetyl-CoA is transferred to histone lysine residues by acetyltransferase, which is necessary for histone acetylation (Shvedunova and Akhtar, 2022). Similarly, lactate serves as an epigenetic substrate for histone lactylation when it is added to histone lysine residues (Zhang et al., 2019; Dai et al., 2022). In 2019, a mass shift of 72.021 Da in histone lysine residues was first identified through a mass spectrometry analysis of MCF-7 cells by Zhang et al., 2019; this shift was comparable to the reaction that occurred when a lactyl group was added to the ε-amino group of a lysine residue. Using the isotope L-lactate (^13^C_3_) in metabolic labeling assays, Zhang et al., 2019 demonstrated that lactate exposure might enhance the lactylation of lysine residues, further supporting the presence of this alteration.

**FIGURE 2:**
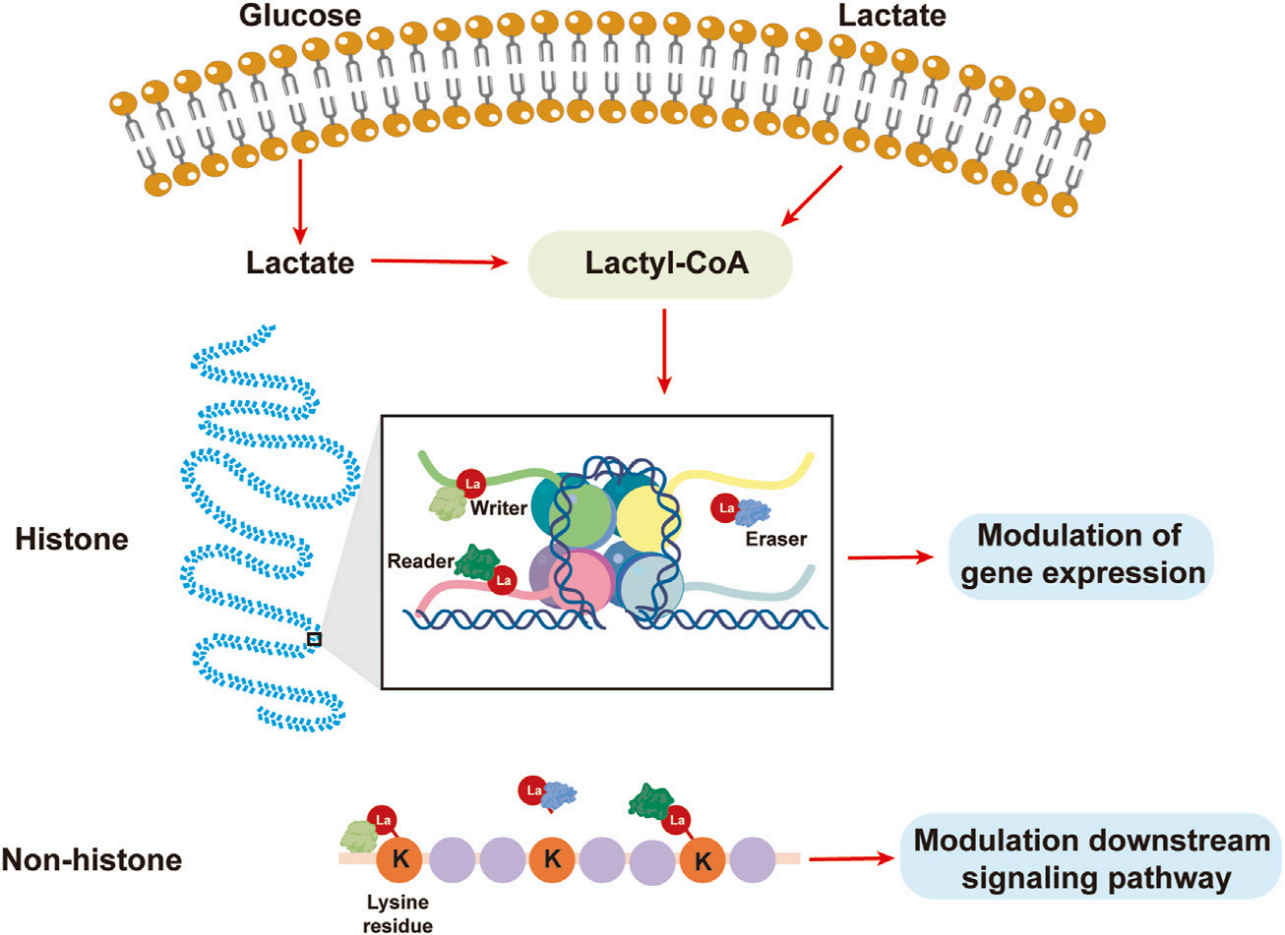
Lactate from the extracellular matrix or glycolysis results in lactylation. Lactate can be used to synthesize lactyl-CoA, after which the lactyl group is transferred by a “writer” to a lysine residue, leading to the lactylation of histones or nonhistones to modulate gene expression or downstream signaling pathways.

According to the results of previous studies, both endogenous and exogenous L-lactate, but not D-lactate, accumulate to a certain point and actively facilitate the lactylation of certain lysine residues (Moreno-Yruela et al., 2022). While mitochondrial inhibitors and cellular hypoxia may increase lactate synthesis and promote lysine lactylation, glycolysis inhibitors are directly correlated with decreased lactate production and lysine lactylation (Liu et al., 2021). The ε-amino group of lysines is responsible for binding the lactyl moieties of lactyl-CoA from L-lactate to the target protein in most of the studied lactylation-modified proteins. This process typically begins with the appropriate enzymes. The lactyl group of lactyl-CoA is first transferred as a substrate to a histone or nonhistone lysine residue by a set of specialized acylases known as “writers,” which change the structure and function of the protein. Then, “erasers,” which function as deacylases to remove some or all of the lactyl groups from the target proteins, emerge to halt the entire lysine lactylation cycle and prevent long-term adverse effects. Finally, this change in lysine lactylation is recognized by effector proteins known as “readers,” which then alter downstream signaling pathways and initiate a variety of cellular processes (Figlia et al., 2020) (Figure 2).

Through overexpression and knockdown experiments, in 2019, Zhao Y et al. showed for the first time that p300 overexpression or interference in HEK293T cells altered the level of histone lactylation. This finding suggested that p300 can function as an acylase, catalyzing the process of histone lactylation. Additionally, transcription and histone modification studies using cell-free recombinant chromatin templates were carried out, and the results showed that the biogenesis of lactylated histones is driven by p300 (Zhang et al., 2019). Research has also revealed low lactylation levels and downregulated profibrotic gene expression in p300-knockdown macrophages (Cui et al., 2021). Similarly, studies have shown that decreasing p300 levels using C646 or interfering with p300/CBP (CREB-binding protein) expression leads to decreased lactylation of high-mobility group protein B-1 (HMGB1) (Yang K. et al., 2022). According to the aforementioned research, p300/CBP may be a “writer” of histone lactylation and may therefore coregulate the incidence of lactylation. The discovery of lactylation offers a new prospective therapeutic target and broadens the traditional concept of research on the carcinogenic mechanism of p300/CBP. Nevertheless, no further lactylases have been discovered, and a thorough description of the precise molecular process underlying the role of p300/CBP as a writer has not yet been published.

Similarly, *in vitro* studies identified the histone deacetylases (HDACs) 1-3 and sirtuins (SIRTs) 1-3 based on the knowledge of other deacylases by methodically assessing the capacity of zinc- and nicotinamide adenine dinucleotide-dependent HDACs. HDAC1-3 has been shown to have robust delactylase activity for both L-lactate and D-lactate, as well as for several short-chain acyl modifications. Furthermore, according to cell overexpression and knockdown studies, HDACs 1 and 3, but not HDAC2, have specialized delactylase activity (Moreno-Yruela et al., 2022). Furthermore, research has shown that several HDAC isomers, including HDAC6 and 8, have the potential to be delactylases. However, the activity of these enzymes is significantly lower than that of HDAC3, whose activity is hundreds of times greater than that of SIRT2 (Zessin et al., 2022). Subsequently, researchers have focused on the role of delactylases in the development of tumors. SIRT2 can prevent the proliferation and migration of neuroblastoma cells by acting as a histone lactylation eraser (Zu et al., 2022). Overall, little is known about lysine lactylation as a new PTM, especially regarding substrates, enzymatic and nonenzymatic modification processes, and lactylation dynamics.

## Lactylation modulates cancer progression

In contrast to those of normal cells, the metabolism of tumor cells “favors” the Warburg effect, resulting in increased levels of lactate in the tumor microenvironment (TME), a crucial tumor characteristic (Hanahan and Weinberg, 2011; Ippolito et al., 2019). High amounts of lactate in the microenvironment are a significant underlying cause of lactylation (Wang et al., 2023), and this finding suggests that there may be a significant increase in lactylation throughout the whole TME, which includes immune cells, stromal cells, and tumor parenchymal cells. Thus, the identification of histone lactylation has opened up new avenues for investigating the function and mechanism of lactate metabolism in the development of tumors, and several unidentified pathways connected to lactylation may play a role in the genesis of cancer.

The mechanism by which histone lactylation controls the growth of tumors is now being elucidated by an increasing number of studies. A favorable TME and its modulation by lactate induce lactylation, which enhances the survival and growth of tumors (Hake et al., 2007; De La Cruz-López et al., 2019; Jin et al., 2022). Lactate-induced lactylation can lead to the recruitment and maintenance of cancer-associated fibroblasts (CAFs), tumor-infiltrating myeloid cells (TIMs; including macrophages, dendritic cells, and regulatory T cells), and cancer stem cells (CSCs) in the TME, in addition to increasing the lactate concentration, which directly shapes the acidic microenvironment and promotes tumor progression and metastasis (Hanahan and Weinberg, 2000).

Lactate itself stimulates vascular endothelial growth factor (VEGF) production and (tumor-associated macrophages) TAM polarization to an M2-like phenotype, both of which aid in tumor growth. Macrophages are activated by tumor-derived lactate signaling through hypoxia-inducible factor 1α (HIF1α), leading to a tumor-promoting state that is marked by increased production of arginase 1 (Arg 1) and VEGF (Colegio et al., 2014). In macrophages, VEGF stimulates the development of new blood vessels, and Arg 1 supplies a substrate for the growth of cancer cells to support tumor growth (Chang et al., 2001; Qian and Pollard, 2010). Further research revealed that under normal oxygen conditions, lactate may stabilize HIF1α through HIF1α lactylation in prostate cancer and subsequently modulate downstream pathways, demonstrating the many roles that lactate and lactylation play in carcinogenesis (Luo et al., 2022). Furthermore, lactate can stimulate the growth of blood vessels and inflammation in an HIF1α-independent manner. Like HIF1α, NDRG family member 3 (NDRG3) is degraded under normoxic conditions in a PHD2/von Hippel–Lindau (VHL)-dependent manner. However, in prolonged hypoxic environments, lactate protects NDRG3 from degradation. This, in turn, causes NDRG3 accumulation, triggers the RAF-ERK signaling cascade, and regulates pathological reactions associated with hypoxia, such as inflammation and angiogenesis (Certo et al., 2021). B-cell adaptor for PI3K (BCAP) stimulates the reparative transformation of macrophages through histone lactylation, and lactate-induced histone lactylation exacerbates this process (Irizarry-Caro et al., 2020; Chen et al., 2021). Recent findings in studies of atherosclerosis (AS) also support the notion that lactylation-mediated macrophage polarization plays a significant role in chronic inflammatory diseases, in addition to tumors. This transition of macrophages from an M1-like to M2-like polarization state converts these cells from a proinflammatory to an anti-inflammatory phenotype (Jin et al., 2021; Yang H. et al., 2022; Xu et al., 2023).

By increasing TGF-β signaling in Treg cells, the lactylation of membrane-organizing extension spike protein (MOESIN) at Lys72 modulates the generation of Treg cells. Patients with hepatocellular carcinoma who have low levels of MOESIN lactylation in Treg cells respond more sensitively to anti-PD-1 therapy. The antitumor impact of combination therapy, including anti-PD-1 and lactate dehydrogenase inhibitors, is greater than that of anti-PD-1 therapy alone, suggesting that lactylation is a very promising target for combination therapy (Gu et al., 2022). In fibrotic lungs, lactylation is upregulated. Mechanistically, lactate causes the histones of macrophage profibrotic gene promoters to become lactylated, which in turn promotes fibrosis (Cui et al., 2021). Studies have indicated a strong correlation between the production of CAFs and tumor-mediated lactate flow in pancreatic cancer, which is intimately associated with the fibrotic matrix. However, further research is needed to determine whether lactylation plays a role in this process (Bhagat et al., 2019). Histone lactylation caused by the Warburg effect stimulates the expression of the NF-κB-related gene LINC01127 in gliomas, which in turn promotes the MAP4K4/JNK/NF-κB axis and glioblastoma cell self-renewal (Li et al., 2023). Furthermore, studies have shown that oxamate suppresses the lactylation of C-C motif receptor 8 (CCR8), a hallmark of Treg cells that infiltrate tumors, hence increasing the effectiveness of CAR-T-cell therapy for patients with glioblastomas (Sun et al., 2023).

Together with other epigenetic changes, lactylation can contribute to carcinogenesis. The YTHDF2 (YTH N6-methyladenosine RNA binding protein 2) promoter region was shown to have increased lactylation signals in ocular melanoma. YTHDF2, an m6A reader, has been reported to serve as an oncogene in a variety of tumors (Paris et al., 2019; Zhang et al., 2020). A poor prognosis for patients with ocular melanoma can result from elevated lactylation levels in tumor tissue because these lactylation levels promote the production of YTHDF2, which promotes oncogenesis (Yu et al., 2021). Lactate-induced lactylation can affect important pathways involved in carcinogenesis and promote the growth and spread of tumors by directly controlling gene expression. In clear cell renal cell carcinoma (ccRCC), lactylation was shown to be elevated due to inactive VHL, which is widely acknowledged as a crucial component in the genesis of ccRCC (Sato et al., 2013). Histone lactylation in ccRCC induces tumor development by stimulating platelet-derived growth factor receptor β (PDGFRβ) signaling. This, in turn, facilitates histone lactylation, creating a positive feedback loop that promotes tumorigenesis in ccRCC (Yang J. et al., 2022). Enterobacterial LPS-induced LINC00152 (ENSG00000222041, CYTOR) has been shown to induce tumor invasion and metastasis in colorectal cancer (CRC) patients. To counteract this effect, LPS upregulates LINC00152 expression by promoting histone lactylation at its promoter, which decreases the effectiveness of binding to YY1 (Wang et al., 2022). Lactylation is a significant factor in tumors, such as non-small cell lung cancer (NSCLC) and hepatocellular carcinoma (HCC).

## Lactate-induced lactylation inhibition as a new strategy in tumor therapy

Currently, an essential tactic to improve the prognosis of patients with tumors is the manipulation of lactate synthesis and transport (Apicella et al., 2018; Spencer and Stanton, 2019; Lin et al., 2022), and the discovery of lactylation has raised the possibility that this modification offers a novel target for halting the spread of cancer and boosting anticancer effects (Fan et al., 2023). Currently, impressive results have been obtained in the clinical application of epigenetic acylation-targeted medications for anticancer therapy. For instance, the FDA has licensed many deacetylase inhibitors, including vorinostat, belinostat, and panobinostat, for the treatment of lymphoma and myeloma (Ogura et al., 2014; O'Connor et al., 2015; Kaufman et al., 2019). The processes of lactate production, transport, or lactylation and the associated effector proteins can all be targeted (Feichtinger and Lang, 2019; Siska et al., 2020; Taddei et al., 2020). Researchers have discovered numerous strong lactate dehydrogenase (LDH) inhibitors, some of which have reached phase I and II clinical trials, that prevent the downstream lactylation process by preventing lactate generation and lactylation; one such inhibitor is oxamate (Allison et al., 2014; Laganá et al., 2019). FX-11, a specific LDHA inhibitor, exhibited anticancer effects in a mouse tumor model and may be a target for cancer treatment (Mohammad et al., 2019). As previously indicated, lactate accelerates the growth of tumors by controlling the lactylation of MOESIN in Treg cells. Additionally, the inhibition of LDHA can effectively decrease both the degree of lactylation and the tumor burden. Furthermore, researchers have shown that individuals who respond to PD-1 monoclonal antibody therapy exhibit a lower level of MOESIN lactylation (Gu et al., 2022). This finding suggests that lactylation alterations might influence the effectiveness of tumor immunotherapy. Moreover, some research has focused on inhibiting the lactylation “writer” p300/CBP or modifying the lactylation “eraser” SIRT2 to effectively manage tumors (Zu et al., 2022; Wang et al., 2023). These studies have led to the proposal of a dual-targeting approach for the treatment of cancer that combines lactate axis targets with immunotherapy or targeted therapy (Davids et al., 2021); however, this therapeutic strategy relies on restricted signal transduction and is still not the best alternative for tumor therapy. Notably, using LDHA inhibitors to treat cancer may present certain obstacles. Disruption of LDHA activity to prevent lactate generation in tumor cells might have uncontrollable side effects. For instance, pyruvate buildup can cause collagen hydroxylation, which promotes ECM remodeling and breast cancer metastasis (Elia et al., 2019).

Previous research has shown that lactate influences gene expression by altering the glioblastoma epigenome, which in turn drives GBM survival. These findings also show that lactate is actively digested in a cellular respiration-dependent manner (Torrini et al., 2022). Thus, targeting oxidative metabolism and lactate metabolism may be a novel therapeutic approach for GBM. However, whether lactate affects the lactylation of histones, which is based on posttranslational histone modifications, can affect gene expression following lactate exposure (Zhang et al., 2019).

Despite mounting evidence showing that lactate is a therapeutic target for slowing the growth of cancer and restoring tumor susceptibility to therapy, whether the precise mechanism of action of lactate is mediated by lactylation is currently unclear. The majority of lactylation-targeting strategies still rely on suppressing lactate production, transport, signal transduction, and even glycolysis. Future objectives should include investigating and determining the “writers,” “erasers,” and “readers” of lactylation modification to properly target lactylation and identifying novel targets for treating gliomas in particular.

## Conclusion and perspectives

With the available treatment options, the median survival of patients with gliomas is currently 15 months, and there has been no discernible improvement in this area over the past 30 years (Gong et al., 2019). The byproduct of anaerobic glycolysis, lactate, is principally exported as lactic acid via MCT4, which increases the pH of the microenvironment around glioma cells. The tumor microenvironment experiences metabolic gradients due to the low extracellular pH, which increases lactate levels. Additionally, high glycolytic flux produces additional metabolites that alter the extracellular matrix and trigger signaling pathways in nearby cells. Growing evidence suggests that lactate serves as a helpful metabolic fuel, a signaling molecule for tumor survival and growth, and a precursor to gluconeogenesis. Furthermore, lactate is involved in the control of the tumor microenvironment and the histone lactylation process, which modifies genes via epigenetic modifications. However, the specific molecular processes underlying these lactylation changes and their significance are unclear. Consequently, it is crucial to provide a thorough explanation of how lactate affects gene expression and epigenetic changes in tumors during the disease course. For the purpose of treating cancer, histone lactylation levels in tumors should be assessed, and the mechanism by which lactylation regulates gene expression should be clarified.

Ultimately, numerous researchers have become interested in the connection between disease states and histone modifications, and understanding how histone PTMs interact with disease modifications will aid the identification of potential therapeutic targets. Over the last 10 years, aberrant expression of enzymes responsible for histone modification has been found in a variety of malignancies (Turner, 2002). For example, the mechanism of action of NCAPG2 is driven by phosphorylated HBO1, which activates H4 histone acetylase and in turn activates the Wnt/β-linked protein signaling pathway, promoting glioblastoma cell malignancy and xenograft tumor growth (Wu et al., 2021). In addition, the sirtuin family of deacetylases has also been found to be involved in glioblastoma (Kunadis and Piperi, 2022). Understanding the pathophysiology of PTMs requires an understanding of their biological features. It is not possible to conduct focused research on antitumor medications targeting PTMs according to their molecular mechanism and advance cancer treatments until the precise mechanism of action of PTMs on tumors is clarified. Therefore, biological research on histone PTMs has great promise for resolving pathological issues and developing novel approaches for cancer prevention and treatment (Liu et al., 2023).
